# Psychotherapy focusing on dialogical and narrative perspectives: a systematic review from qualitative and mixed-methods studies

**DOI:** 10.3389/fpsyg.2024.1308131

**Published:** 2024-08-06

**Authors:** Augusto Mellado, María Teresa del Río, Paola Andreucci-Annunziata, María Elisa Molina

**Affiliations:** ^1^Facultad de Medicina y Ciencias de la Salud, Universidad Central de Chile, Santiago, Chile; ^2^Facultad de Medicina y Ciencias de la Salud, Universidad Mayor, Santiago, Chile; ^3^Laboratorio Transformación y Agencia Humana, Instituto de Bienestar Socioemocional, Facultad de Psicología, Universidad del Desarrollo, Santiago, Chile

**Keywords:** dialogic, narrative, subjectivity, intersubjectivity, change in psychotherapy, systematic review

## Abstract

**Introduction:**

This systematic review identified qualitative and mixed-methods empirical studies on psychotherapy from dialogical and narrative approaches, aiming to address the following questions: (1) How are subjectivity and intersubjectivity qualitatively understood in dialogical and/or narrative psychotherapies studied using dialogical and narrative approaches? (2) How do therapeutic changes occur, including their facilitators and barriers? (3) What psychotherapeutic resources are available for psychotherapists in these types of studies?

**Method:**

The articles were selected according to the Systematic Reviews and Meta-Analyses (PRISMA) guidelines and the eligibility criteria proposed by the PICOS strategy (participants, interventions, comparators, outcomes, and study design) from 163 records identified in the Web of Science Core Collection databases.

**Results:**

The systematic review process allowed the selection of 16 articles. The results provided insights into the understanding of subjectivity, intersubjectivity, change in psychotherapy, its facilitators, and barriers from these perspectives. It also offered some therapeutic interventions that can be implemented in psychotherapies, integrating dialogical and/or narrative aspects.

**Discussion:**

The centrality of dialogical exploration of patient/client resources, therapists as interlocutors fostering client agency, polyphony serving as scaffolding for change, and interconnection with the sociocultural environment are discussed. The integration of this latter topic has been a challenge for these types of studies, considering the active construction of shared meanings. The dialogical and narrative approaches focus psychotherapy on transforming meanings through dialogue and re-authoring stories, evolving within cultural and historical contexts. Thus, this study highlights the relevance of these perspectives in contemporary psychotherapy, emphasizing dialogue in co-creation within an intersubjective framework.

## 1 Introduction

The evolution of psychotherapeutic knowledge includes the integration of approaches that have been proposed at other times or from other disciplines and that allow for resituating concepts from a broader and more contemporary perspective. Certain psychological paradigms that extend their fields of action to psychotherapy have taken up notions coming from, among others, linguistics, anthropology, or the classics of psychology, sustaining a form of understanding that is usually comprehensive, as it is complemented and integrated by different lenses, such as the semiotics and cultural studies of R. Barthes, the historical-cultural psychology of Lev Vygotsky, the literary theory, sociolinguistics, and culturalism of M. Bakhtin, or the functionalism of W. James (Hermans and Gieser, 2013a; Valsiner, 2014).

In general, some of these new traditions recognize that, in any psychotherapy, an interaction is established between at least two individuals, in which patients/clients construct stories in the presence of themselves and their therapists. These stories are constructed within an exchange or dialogical space in which novel content or new connections between their constituent parts can be established (Hermans, 2004). Furthermore, this reflects the activation of narrative processes by patients/clients, shaping these specific stories or accounts and giving them structures that convey other contextualizing aspects, imparting a narrative quality to them (McLeod, 1997). Psychotherapy would then be a dialogic scenario in which stories are told and actualized, sometimes as descriptions and other times as narratives, drawing on their constituent elements from the constant interaction of the patient's self, the therapist, and the cultural and historical contexts in which they are embedded. Narratives are forms of organization of different experiences in which the narrators are storytellers and that may include themselves, that is, in self-narratives organized within an intersubjective and dialogical process in which the therapist and patient/client intertwine internal and external, real and imaginary dialogues (Hermans and Hermans-Jansen, 1995).

In recent decades, two approaches have stood out for their contributions to understanding dialogic and narrative construction in psychotherapy. One is rooted in the dialogical perspective, primarily, though not exclusively, in dialogical self-theory (Hermans and DiMaggio, 2004; Konopka et al., 2018a), and the other is known simply as narrative therapy (White and Epston, 1990; Angus and McLeod, 2004). The dialogical perspective integrates a modern understanding of patients' selves, considering coherence and continuity, with a postmodern or post-Cartesian perspective suggesting disorganization, fragmentation of the self, and transcending external/internal duality (Hermans, 2003, 2014). Both dialogical and narrative approaches reference constructivist and constructionist thinking, conceptualizing identity as multiple, changing, and interpenetrating with the social world (Hermans et al., 1992; Neimeyer, 2006).

There are also dialogical and narrative contributions to individual and family psychological assessment methods. At the individual level, collaborative assessment methods (CAM) promote the involvement or collaboration of patients in their evaluative processes through a procedure of triangulating narratives (Aschieri, 2012), co-constructing assessment goals and tasks in coherence with their life experiences and goals (Aschieri et al., 2023), which will ultimately influence more integrated self-narratives and therapeutic change. At the family level, the Therapeutic Assessment with Families with Children (TA-C) proposes that test results be addressed as hypotheses that can be discussed and reconstructed among the family and the evaluators, fostering flexibility and the development of creative solutions to difficulties (Aschieri et al., 2012).

The dialogical approach proposes that the self is shaped by a set of I-positions that function as a society of the mind capable of conceiving dialogical or monological interactions (Konopka et al., 2018b). Based on the Bakhtinian notion of speech genres, which are defined as heterogeneous temporal structures of discourse and as relatively stable types of utterances that settle in each sphere of language use (Bakhtin, 2022), for dialogism, there exists no discourse outside the speech genre. Psychotherapy can be understood as a particular cultural practice organizing discursive exchanges between therapist and patient, considering it a language community or part of situational languages (Leiman, 2011; Martínez et al., 2014). Moreover, similar to a polyphonic novel, each self-position can construct specific narratives through a multiplicity of voices (Hermans, 1996, 2003). The voices engage in a dialogue between internal and external I-positions, with the external referring to positions where others are present within the I, that is, the other conceived as another I within an extended dialogical self (Hermans, 2014). It is also possible for certain external positions to be internalized by adopting others' viewpoints (DiMaggio et al., 2010). Thus, an I-position can be considered the place from which voices engage in internal dialogue or emerge toward the external world. Seikkula (2008) asserted that experience leaves traces in the body, but only a minimal portion is formulated into spoken narratives. When converted into words, they become voices. Voices are also defined as traces or marks that are activated and triggered by new events similar to or related to an experience (Stiles, 2002). Therefore, individuals would have multiple internal voices. These inner voices can be understood as sub-communities of experiences connected through shared and mutually accessible meanings (Osatuke and Stiles, 2006), imbued by broader social and cultural contexts. In dialogical exchanges, voices are spatially positioned, and narratives are temporally displayed (Pollard, 2019).

Similar to other postmodern therapies, the dialogical perspective understands change in psychotherapy as a semantic process of transforming meanings (Avdi and Georgaca, 2007), as can be seen in the narratives that emerge from dialogical exchanges between therapists and patients/clients. The transformation of meaning is based on the negotiation and exchange of signs between the I and the other, which are socially acquired in the processes of semiotic mediation (Valsiner, 2002; Salgado and Clegg, 2011). The dialogical self can generate self-reflective processes that accompany its transition from monological and rigid to dialogical and flexible discursive exchanges. These processes can be supported by positions called promoters and meta-positions. Promoter positions have the potential to generate and organize more specific, future-oriented positions and contribute to the reorganization of the self. On the other hand, meta-positions allow for maintaining some distance from other positions and provide an overview that allows the observation and identification of significant connections between them (Hermans and Gieser, 2013b). Changes are based on the multivoiceness in dialogue that constructs new meanings as new conversations are established (Rober, 2005; Seikkula and Arnkil, 2019), including therapeutic conversations that enhance novel distinctions in stories and narratives. Narrative therapy considers it fundamental that the organization of the life events of patients/clients revolves around specific stories or narratives expressed in sequences over time, allowing for the formation of coherent narratives of themselves and the world around them (Neimeyer, 2000; Adler, 2013). The representations of these stories or narratives, which are based on experiences, determine how life and interpersonal relationships are experienced. White and Epston (1990) suggest that stories have a characteristic of “relative indeterminacy” or ambiguity due to the presence of implicit meanings, varying perspectives, and a diversity of metaphors that can enrich event descriptions. They would present states of credibility that point not to a final state of certainty but to varying perspectives. Their morphosyntactic structure is organized in relation to time and often manifests grammatically in the subjunctive mood (expressing actions, states, or situations that are hypothetical, possible, uncertain, or subjective) instead of an indicative mood (expressing actions, states, or situations considered real, objective, and concrete).

Therapy from this approach focuses on “therapeutic conversations” as it transcends the linearity of an intervention or treatment. The general assumption is that people face problems and seek therapy when the narratives they construct about their experiences do not adequately represent those experiences. The therapeutic conversations allow patients/clients to disengage from the dominant stories that have influenced their lives and relationships. The externalization process promotes the interpretation of new meanings by integrating novel outcomes into alternative stories that can be coherently integrated. As storytellers, patients/clients can position themselves as protagonists in their experiences. Based on the notion of interpretive acts, the retelling of stories as novel narratives becomes relevant either by themselves or together with others, engaging in the constant re-authoring of one's own life and interactions with others. In doing so, they may discover important aspects of their experience that they had not previously considered but that may potentially be significant (White and Epston, 1990; McLeod, 2006; White, 2016).

One common and possibly core aspect of the dialogical approach and narrative therapy is the concept of self-narratives. Any process of “reconstructing subjectivity” (Avdi and Georgaca, 2007) requires a transformation in the self-narratives, whether it is understood as a re-authoring or a re-subjectivation process. A self-narrative consistent with lived experiences allows for a sense of continuity and meaning, organizing life in the present moment and interpreting subsequent experiences (White and Epston, 1990). Therapeutic conversations can facilitate integration and greater awareness among the multiple voices and positions of patients/clients (Martínez et al., 2014) to the extent that the dialogues integrate self-narratives into their stories or narratives. The generation of therapist-patient/client dialogues that enable these changes requires the emergence of an intersubjective awareness in which the issues raised can be addressed by paying attention to and suggesting possible new perspectives on them (Seikkula and Arnkil, 2019). The relational perspective underlying these approaches considers subjectivity to be the result of internal dialogues and allows us to understand intersubjectivity within the framework of communicative relations intertwined with culture and social institutions (Rober et al., 2008; Gillespie and Cornish, 2010). According to Marková (2003, 2016), the dialogical perspective on intersubjectivity could be situated in a dimension of irreducible interdependence between self and other(s) in which experiences are constituted in a given social and cultural context. According to the author, from a Bakhtinian perspective, this inter-subjective dynamic is better expressed by the concept of co-authorship. In this way, dialogical interaction would not result in the fusion of self and other(s) but rather in an active understanding of the unfamiliar within oneself in reference to others, in discourse (leading to tension between Ego and Alter), and in the result of their own positions arising from this exchange or confrontation. This struggle for the construction of meanings is established in a frontier in which the limits are diffusely demarcated by the interrelationship between self and other (s) (del Río, 2012), who are co-authors and share responsibility for the communication processes they establish.

A critical review of narrative research in psychotherapy included 20 articles (published between 1997 and 2005) and identified four specific aspects: (a) studies that produce thematic analyses of the content of narratives, (b) studies that produce typologies of client narratives, (c) studies that employ a dialogical approach to the study of narratives, and (d) studies that focus on narrative processes (Avdi and Georgaca, 2007). Within the scope of the two dialogical studies collected by this review (aspect c, including two studies in total), it can be observed that therapeutic change can be evidenced in the development of richer dialogues among the main characters in the patients'/clients' narratives as well as in the development of a reflective and observational metaposition.

A meta-synthetic systematic review that collected 35 articles (published between 1992 and 2018) based on systemic and constructivist approaches in psychotherapy addressed the process of change in therapeutic dialogue in sessions and found four main themes: (a) shifting to a relational perspective, (b) shifting to a non-pathologizing therapeutic dialogue, (c) moving dialogue forward, and (d) the dialogic interplay of power (Tseliou et al., 2021). Regarding the dialogical studies included in this systematic review, it was possible to establish results in three of the themes that emerged (themes a, c, and d, including four studies in total), leading to the conclusion that the evolution of psychotherapy requires a shift from monologic to dialogic and an alternation in the distribution of the exercise of power. These dimensions point to the achievement of polyphony and communicative reflexivity based on therapists' receptivity and authenticity in relation to the voices of patients/clients, using techniques such as open-ended questions, cross-voice positioning (adopting the voices of others), or the introduction of alternative meanings.

These two reviews address important aspects of the dialogical and narrative understanding of psychotherapy studies. However, both studies have limited scope with respect to the number of properly dialogical studies reviewed and do not include in their search any research based on methods built from these perspectives and applicable to psychotherapy, such as the Personal Position Repertoire (PPR) (Hermans, 2001, 2006), Narrative Process Coding System, or Narrative-Emotion Process Coding System (Angus et al., 1999, 2017), among others. A third recent review was built exclusively on studies designed from the latter two aforementioned narrative coding methods and concluded that these coding systems allow the recognition of whether patients/clients are in non-productive processes, such as identifying impersonal and superficial narratives (problem markers), or whether they are in the process of change (transition markers) (Aleixo et al., 2021). These systems enable distinguishing between different ways of constructing narratives, aiding in understanding the stages of the change process, and guiding potential interventions.

Unlike previous reviews, the present systematic review aimed to identify studies based on qualitative methods in psychotherapy that clearly presented a dialogical and narrative perspective in the intervention offered, design, and interpretation of results. The questions addressed in the current systematic review were as follows: (1) How are subjectivity and intersubjectivity qualitatively understood in dialogical and/or narrative psychotherapies studied using dialogical and narrative approaches? (2) How do therapeutic changes occur, including their facilitators and barriers? 3. What psychotherapeutic resources are available for psychotherapists in these types of studies?

## 2 Materials and methods

The Preferred Reporting Items for Systematic Reviews and Meta-Analyzes (PRISMA) guidelines (Page et al., 2021a,b) were used for this review, and the PICOS (Participants, Interventions, Comparators, Outcomes, and Study Design) strategy was used to establish the eligibility criteria for the studies (Higgins and Green, 2008; Methley et al., 2014). According to the checklist of the PRISMA guidelines (Page et al., 2021a,b), the following quality steps for systematic reviews were verified in line with the following sections: 1 (title), 2 (structured abstract), 3 (rationale), 4 (objectives), 5 (eligibility criteria), 6 (sources of information), 7 (search strategy), 8 (selection process), 9 (data extraction process), 10a and 10b (data items), 11 (study risk of bias assessment), 13 (synthesis methods), 16a and 16b (study selection), 17 (study characteristics), 18 (risk of bias in studies), 23 (discussion), 24 (registration and protocol), 25 (support), 26 (competing interests), and 27 (availability of data, code, and other materials). The following sections were excluded because the data from each study that satisfied the criteria were not considered pertinent for the present review, were not available, or were presented only in a general manner after having been part of a respective protocol: 12 (effect measures), 14 (reporting bias assessment), 15 (certainty assessment), 19 (results of individual studies), 20 (results of syntheses), 21 (reporting biases), and 22 (certainty of evidence). This systematic review was registered with PROSPERO (CRD42023418839) and is available at https://www.crd.york.ac.uk/prospero/display_record.php?ID=CRD42023418839.

The search strategy and selection of studies that are part of the review are described below and are analyzed following the guidelines for narrative syntheses in systematic reviews suggested by the document PRISMA-P 2015 (Popay et al., 2006; Shamseer et al., 2015). The choice of narrative synthesis rather than qualitative meta-analysis or meta-synthesis was based on the descriptive nature of the information sought from the studies and the different potential methodological sources used to create dialogical and narrative data. In contrast, the criteria for guiding qualitative meta-analyses or meta-syntheses (Walsh and Downe, 2005; Levitt, 2018) require diverse studies that delimit methodological techniques (in our case, for example, we could include phenomenological and hermeneutic methodologies) and similar thematic issues or types of studies, which can be compared (in our case, we will include naturalistic studies as well as single and multiple case studies), thus avoiding, in this particular case, problems with the reliability of the results.

### 2.1 Search strategy

A group of articles was established as a homogeneous citation base, thereby safeguarding the independence of indexing databases that use different calculation strategies to determine the impact factors and quartiles of the journals (Bakkalbasi et al., 2006; Falagas et al., 2008; Chadegani et al., 2013; Harzing and Alakangas, 2016; Mongeon and Paul-Hus, 2016). The review relied on the Web of Science (WoS) core collection, selecting articles published in journals indexed by WoS via a search vector on this topic in psychotherapy [TS = ((psychother^*^) AND ((dialogic^*^) OR (narrati^*^))] without restricted temporal parameters. The extraction was performed on 5 April 2023. Only documents typified as articles by WoS were included, regardless of whether they had additional parallel typifications by WoS. Next, considering the focus of this review, the key term “qualitative” was applied to each abstract of the pre-selected articles to filter out studies that did not explicitly declare the use of this methodology, which is predominant in dialogical and narrative studies.

### 2.2 Eligibility criteria

The articles were selected based on the PICOS eligibility criteria (Table 1).

**Table 1:** Eligibility criteria using Participants, Interventions, Comparators, Outcomes, and Study Design (PICOS).

**PICOS**	**Description**
Participants	Children, adults, couples, families, or groups of patients in psychotherapies are analyzed from dialogical and/or narrative perspectives, or where reference is made to clinical material derived from these processes. Therapists may be included as long as therapeutic processes with patients are considered
Interventions	Psychotherapies and clinical interventions (e.g., counseling) or groups of interventions following dialogical and/or narrative models
Comparators	Distinction of outcomes according to roles of participants, success/failure of psychotherapy, and qualitatively distinct segments/excerpts within psychotherapy, when applicable
Outcomes	Psychotherapy outcomes (e.g., wellbeing, changes in narrative identity, transformation of subjectivity) can be investigated using the qualitative methods employed in the studies
Study design	Studies with qualitative or mixed-methods designs, including grounded theory, thematic analysis, dialogical analysis, phenomenological approaches, narrative perspectives, and constructivist and socio-constructivist perspectives

### 2.3 Study selection and data extraction

First, duplicate articles were manually removed. Review articles, early access papers, proceeding papers, editorial material, book reviews, book chapters, meeting abstracts, books, corrections, discussions, letters, and reprints were excluded from the analysis. Articles not related to psychology research were also excluded. Finally, articles that were not in English were also excluded.

Afterward, the titles and abstracts of each article were checked for relevance by all authors of this work (clinical psychologists with postgraduate dialogical training). Subsequently, the authors independently reviewed the abstracts and full texts of potentially eligible articles using the PICOS criteria. The eligibility stage leading to the final inclusion was first independently conducted by the four authors, with an equal distribution of documents listed in an Excel spreadsheet from the previous stages. During this procedure, any disagreements were addressed and discussed by the two authors and were consistently ensured through intersubjective consensus (Levitt et al., 2021).

### 2.4 Quality assessment and risk of bias

The Mixed Methods Appraisal Tool (MMAT) was used to assess the quality and risk of bias of the included studies. The MMAT scale is a valid measure of the methodological quality of the article. All of the authors independently applied the instruments to each selected study.

MMAT includes a checklist based on the synthesis of qualitative and quantitative evidence, including criteria for the evaluation of mixed studies for systematic reviews. It defines the study category, and seven items are applied according to a score from zero to one to obtain a final percentage mean. Studies were considered high (>75%), moderate (50%−74%), or low (< 49%) quality (Hong et al., 2018; Arenas-Monreal et al., 2023).

## 3 Results

The search over an unrestricted period in the WoS main collection resulted in 2,688 documents from eight different databases in the Web of Science Core Collection (SSCI, Social Sciences Citation Index; SCI-EXPANDED, Science Citation Index Expanded; ESCI, Emerging Sources Citation Index; CPCI-SSH, Conference Proceedings Citation Index—Social Science and Humanities; CPCI-S, Conference Proceedings Citation Index—Science; BKCI-SSH, Book Citation Index—Social Sciences and Humanities; BKCI-S, Book Citation Index—Science; A and HCI, Arts and Humanities Citation Index). Excluding records according to document type (625), non-psychology research area (*n* = 624), and non-English-language articles (257) resulted in 1,182 records published between 1992 and 2023 for screening (details in Supplementary Table 1). Applying the “qualitative” key term to abstracts, 1,019 articles were excluded, reducing the corpus analyzed to 163 full-text articles retrieved and screened using the selection criteria defined with the PICOS strategy. Finally, articles that presented studies not based on psychotherapeutic empirical qualitative studies using dialogical and narrative approaches (147) were excluded. Ultimately, 16 studies published between 2003 and 2023 were selected and evaluated for quality.

**Figure 1:**
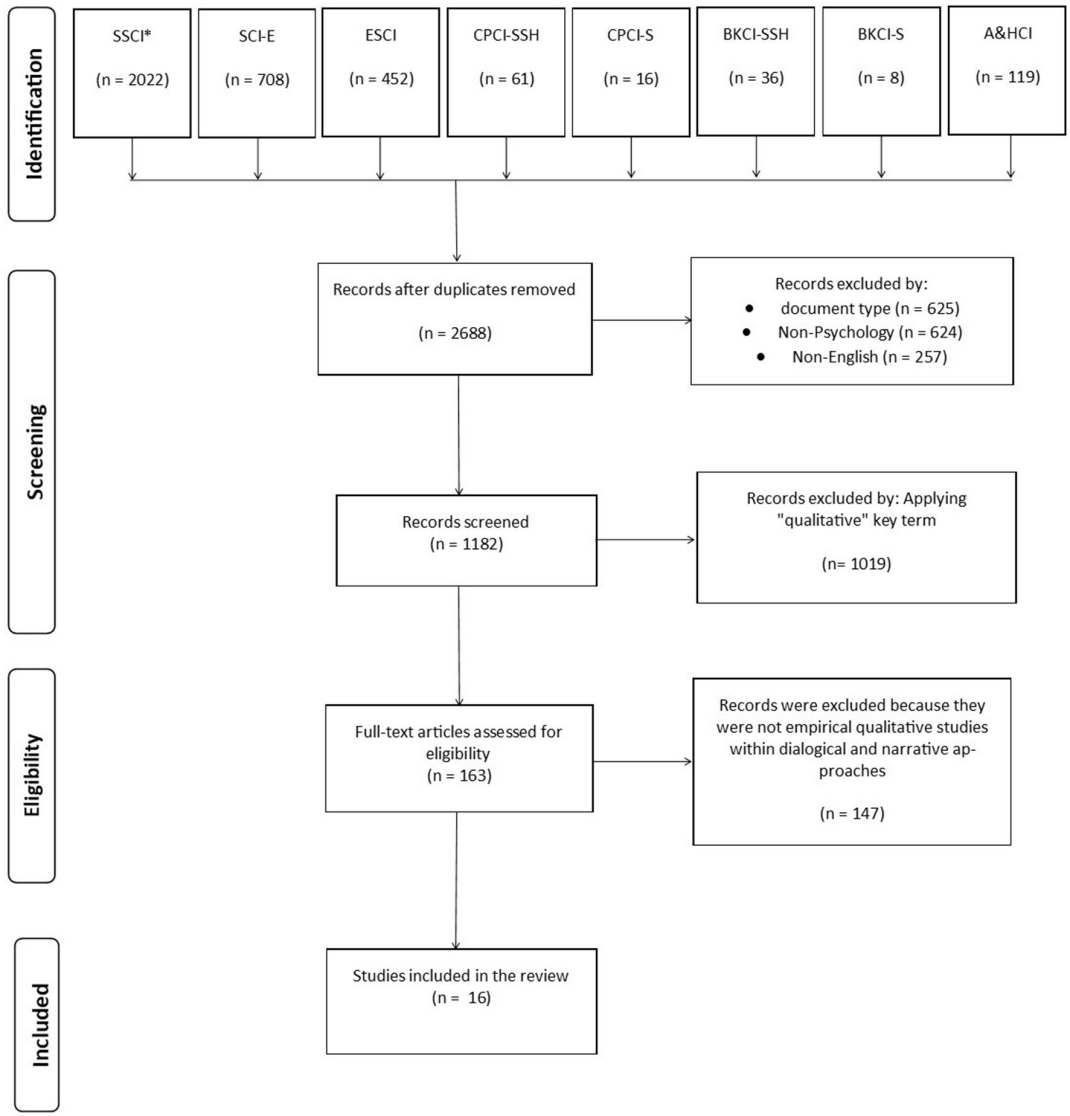
PRISMA diagram flow. *SSCI, Social Sciences Citation Index; SCI-EXPANDED, Science Citation Index Expanded; ESCI, Emerging Sources Citation Index; CPCI-SSH, Conference Proceedings Citation Index—Social Science and Humanities; CPCI-S, Conference Proceedings Citation Index—Science; BKCI-SSH, Book Citation Index—Social Sciences and Humanities; BKCI-S, Book Citation Index—Science; A&HCI, Arts and Humanities Citation Index.

The document set entered as input in the PRISMA diagram flow according to the eligibility criteria (PICOS) listed in Table 1 is shown in Figure 1.

A summary of the general characteristics of the included systematic reviews is presented in Table 2.

**Table 2:** Characteristics of the reviewed studies.

**References (country)**	**Participants (*N*)**	**Interventions (techniques and methodologies)**	**Comparator**	**Results**	**Study design**
Kay et al. (2023) (UK)	Five patients ranged from 20 to 30 (three men and two women). Four therapists ranged from 28 to 48 (1 male, three female)	Transcripts from the first, middle, and final psychotherapy sessions of five individuals treated for depression, all showing reliable improvement, were analyzed Qualitative method of analyzing multivoicedness (QUAM)	Not applicable	Two voices were identified in patients after treatment. Opposing voices during depression are submissive and assertive, agentic, and reflecting voices after treatmentA third one can be used to develop treatments that can identify inner conflict and suppression and cultivate reflexivity in patients	A qualitative descriptive study
Chiara et al. (2023) (Italy)	Five female patients ranged from 20 to 30	Narrative psychotherapy journey. A thematic analysis was conducted on the transcripts of the therapeutic pathways, highlighting the positions of the self-identified Dialogical self-theory qualitative analysis of psychotherapy sessions	Not applicable	Two main themes in the narratives facilitating PTG (post-traumatic growth) were identified, and three in the narratives acted as barriers there, too	A qualitative descriptive study
Hills (2023) (UK)	Four patients, 1 therapist	Multicase study of the researcher's own clinical work Autoethnographic account in which the researcher uses supervision, personal therapy, dreams, and life events Sessions were audio-recorded and coded for qualitative markers indicating the emergence of novel self-narratives within a narrative-dialogical framework	Not applicable	The study highlights the use of self as a research instrument and offers a candid and intimate example of how practitioner research might be structured and delivered	A qualitative descriptive study
DiMaggio et al. (2003) (Italy)	Two patients (one male aged 15, one female aged 25)	Analysis of a clinical case to show how a patient put together her set of narratives using the tool of self-investigation through writing a diary Qualitative microanalysis of the diaries written by the patient during psychotherapy	Difference in the intensity of negative emotion expression between the beginning and end of therapy	The patient's negative emotions diminished in intensity, and guilt feelings gave way in the texts to better narratives	A qualitative descriptive study
Mellado et al. (2022b) (Chile)	One female patient is aged 53 years, and one male therapist is aged 31 years	Dynamic patterns were identified, and the hypothetical attractors (i.e., the most stable patterns in the interaction of voices) were defined using the Space State Grid (SSG) Voices and personal positions were identified by the Model of Analysis of Discursive Positioning in Psychotherapy (MAPP)	Different stages of psychotherapy	Differentiated dynamic patterns of voices were identified depending on the stage of psychotherapy Subjective transformation from a monological to a dialogical dimension, a transition from a state of dissociation of the patient to a reorganization of her subjectivity	A mixed-design case study
Mellado et al. (2022a) (Chile)	One female patient is aged 53 years, and one male therapist is aged 31 years	Change episodes of the patient were traced through the Change Episodes Model Dynamic patterns of voices were identified by the Space State Grid (SSG) Voices and personal positions were identified by the Model of Analysis of Discursive Positioning in Psychotherapy (MAPP)	Change episodes of different types of subjective elaboration	The results established differentiated dynamic patterns in the patient's change episodes	A mixed-design case study
Steen et al. (2023) (Netherlands)	15 life stories of patients	Seven themes were selected from patients diagnosed with personality disorders Investigate meaning in life in 15 life stories of patients with PD before and after intensive psychotherapy Qualitative narrative analysis uses a holistic content approach and theoretical thematic analysis	Pre-treatment life stories and post-treatment are presented in seven theoretical themes	Pre-treatment and post-treatment comparison of themes The presence and/or emergence of specific meaning in life themes could result from attaining treatment goals that ameliorate personality dysfunction The results are proposed as an alternative to the DSM-5 as a model for personality disorders	A qualitative descriptive study
Dawson et al. (2021) (Australia)	13 women (six service users and seven practitioners)	The psychotherapy context is based on Open Dialogue (Seikkula et al., 2003) training, where practitioners receive regular Open Dialogue supervision Qualitative interviews	Not applicable	The findings suggested that dialogical processes created safety by attending to multiple voices in nonviolent ways that reduced perceived hierarchies. Notions of expertise were renegotiated, which allowed the women to feel heard in significant ways that were different from their previous experiences with other social and healthcare services	A qualitative descriptive study
Kay et al. (2021) (UK)	One patient (30-year-old) male	Patient Health Questionnaire (PHQ-9) Generalized anxiety disorder 7 (GAD-7) Qualitative method for analyzing multivoicedness (QUAM)	Not reported	QUAM permitted a close inspection of the individual in their social context through an examination of the dialogs that were generated around a range of internal and external I-positions Two prominent opposing internal I-positions appeared to dominate: I-as-monitor (patient observed and monitored by himself) and I-as craving (no control over his food consumption) I-as-repulsive (had a strong emotional valence), and I-as vulnerable (had a gentler and more sensitive quality, and a pensive, thoughtful delivery)	A mixed-design case study
Råbu et al. (2019) (Norway)	Six therapists-researchers	Collective autoethnography	Not applicable	Negative experiences could strengthen one's own convictions for acting differently; positive experiences worked as inspiration and support; being in therapy early in life represented a significant formative experience; and working through complex personal issues in therapy gave the courage to identify similar conflicts in the fantasies and realities of clients	A qualitative descriptive study
Penttinen et al. (2016) (Finland)	Female patient (~40 years old); male therapist (~50 years old) The Assimilation of Problematic Experiences Scale (APES)	Assimilation analysis (Honos-Webb et al., 2003)	Not applicable	Progress was found in the patient's assimilation process when she adopted a reflective stance regarding her internal experience or external actions. Reflexivity manifests in different forms and at different levels. The therapist's responsiveness facilitated the patient's increased reflexivity and her progress in assimilation	A qualitative descriptive study
Cardoso et al. (2016) (Portugal)	Four adolescent patients, 18 years old (one male, four females)	Interpersonal Process Recall Inquiry Interviews (Kagan, 1975) Thematic analysis is a theory-driven process (Watson and Rennie, 1994)	Not applicable	Four basic sequential operations of meaning construction were identified in the patients: symbolic representation of experience, making new realizations, reflexive self-examination, and revisioning self. Additionally, during the negative moments of the sessions, they described feelings of vulnerability, and during the positive moments, feelings of security	A qualitative descriptive study
Piazza-Bonin et al. (2016) (USA)	Three adult female patients and three male therapists [a commercially distributed video series created by the American Psychological Association (APA)] Global Assessment of Functioning (GAF)	Innovative Moments Coding System (Gonçalves et al., 2011) Segmented Working Alliance Inventory–Observer-Based Measure (S-WAI-O)	Not reported	The patients maintained a good alliance and achieved satisfactory overall outcomes The results highlight the presence of innovative moments of reflection and reconceptualization in the reconstruction of meanings in these grief therapies	A mixed-design case study
Boothe et al. (2010) (Switzerland)	Twenty-three sessions with a female patient. The process lasted for 61/2 years, with a total of 326 sessions	Narrative Analysis: Examining and deconstructing patients' narratives to gain insights into their psychological processes, conflicts, and coping mechanisms JAKOB Narrative Analysis (Boothe, 2004): Systematic interpretation of narratives in psychotherapy. Focus on the communicative category “narrative.” The analysis can consider neither the therapist's comments nor the interaction The transition from textual analysis to the development of psychodynamic hypotheses represents a shift from the construction of the narrative to the exploration of psychological regulation	Not reported	Narratives and conflicts between desires and defenses Characteristic desire/anxiety/defense movements of the patient/narrator are identified, compared, and combined for a broader interpretation In the final phase of therapy, significant changes in narrative dynamics are observed. The initiative for action is concentrated in the ego, which also holds a central position, while other actors are marginalized. Defense mechanisms reveal that the ego does not handle the oedipal desire, confronting it with childish anxieties and distancing it	A qualitative case study
Danner et al. (2007) (USA)	Fourteen women were referred to the therapy group. The average age of the participants was 42.6 years	Brief Interview. An 11-item interview was conducted during the initial screening session A Hmong adaptation of the Beck Depression Inventory The main technique was an exploratory focus group. The questions focused on the women's perceptions of their concerns and problems and their expectations for the therapy group. Post-treatment focus group questions assessed the women's experience of the therapy group	Not reported	The intervention was effective in helping Asian participants gain social and emotional support, identify coping mechanisms, and integrate Western practices with those from their own culture Positive therapy feedback: (1) getting out of the house; (2) discussions with other women and meetings with friends; (3) assistance with citizenship applications; (4) learning relaxation techniques; (5) calling others for support; (6) advice and education on coping skills; and (7) identification of lasting coping mechanisms Negative feedback: (1) positive effects limited to time in a group; (2) still struggling with depression and physical symptoms after completing the group; and (3) 10 sessions are not enough	A mixed descriptive study
Pote et al. (2003) (UK)	Five senior family therapists from the LFTRC (Leeds Family Therapy and Research Center) in the UK Fifteen videotapes of therapy sessions were purposefully sampled from the library of therapy sessions held at LFTRC	Semi-structured interviews with therapists. The Brief Structured Recall (BSR) method was adapted for the interviews, requiring therapists to review and comment on videos of their own practice Videotapes were selected from the beginning, middle, and end sessions of therapy. These were observed while looking to rate (1) the therapist's intention, (2) the family trigger event, (3) the therapist's techniques, and (4) the content of the discussion Grounded Theory (Strauss and Corbin, 1990)	Not applicable	It is possible to manualize TFS (functional family therapy), taking care of methodological and ethical aspects Regarding the objectives, therapists used different interventions, using linear and circular questions to understand perspectives. For complex goals, circular questions and statements were used to distinguish the ideas of the team and therapists Notable differences were observed in therapist and family activities at different stages of therapy, allowing the structuring of prescriptions in the manual for opening, middle, and closing sessions Therapists showed high adherence to the manual prescriptions in the questionnaire and in the mid-sessions	A qualitative descriptive study

The proposed research questions were addressed using a narrative synthesis procedure applied to the selected articles. A summary of the definitions of subjectivity, intersubjectivity, change in psychotherapy, facilitators of and barriers to change, and the main therapeutic interventions from dialogical and narrative approaches obtained from each of the studies can be found in Table 3, while Table 4 shows the salient qualitative methodologies and techniques arising from each of the articles in the present review.

**Table 3:** Main disciplinary findings in the studies.

**References (country)**	**Subjectivity definition**	**Intersubjectivity definition**	**Psychotherapeutic change**	**Change facilitators/change barriers**	**Therapeutic interventions from dialogical/narrative models**
Kay et al. (2023) (UK)	The self is conceptualized as a multiplicity of interacting voices engaged in communicative interchange	The internalized psychotherapist, in the form of an external I-position, becomes bonded to an already existing I-position. This strengthens the external I-position's abilities within the repertoire, as it becomes a hybridized version of its previous self	Longitudinal changes in the pattern of I-positions	Experiencing emotion—in the form of emotional I-positions manifested in psychotherapy—may enable subsequent growth in reflexive capacity. Growth of the emotional I-positions, which may facilitate an increase in the breadth of dialogicality, leading to further integration On the other hand, monological organization and the absence of dialogue between I-positions, coupled with the dominance of the suppressive I-position, can be understood as hindering reflexive growth and the development of a meta-position	To promote meta, I use positions that reflect and facilitate dialogue Empathic awareness of the nature of the client's psychological boundaries. These intrapersonal processes could be understood as the relations between external I-positions and internal I-positions Offering empathy to all I-positions may encourage each of them to be manifest and thereby open to transformation during the psychotherapy process
Chiara et al. (2023) (Italy)	The self as a narrative organization The self is relational The self is inhabited by a multiplicity of selves and “voices.” The inner world is seen as organized, starting with the social and relational world in which they take part	Human beings are relational and inhabited by a multiplicity of selves; their stories are no longer seen as a unit but as multiple and are socially constructed in dialogue with other people	New narratives and counter-narratives to dominate ones of victimization	Shared emotional experience Not sharing narratives in meaningful emotional contexts	Explore ways to generate a dialogue with other I-positions expressing an alternative or counter-narrative Participating in social events and dealing with an experience in a shared, emotionally dense context can promote PTG (post-traumatic growth) The therapist manages the I-position of “I, the victim” and the victimizing narratives by listening and accepting, allowing the dominant I-position of victimization to be expressed Therapists can explore ways to generate dialogue with other I-positions expressing an alternative or counternarrative
Hills (2023). (UK)	Self-conceptualized as moving I-positions mobilized by other internal and external voices	Intersubjectivity implies purposefulness and directionality in the therapist's response to clients as a consequence of holding a coherent model of change	An evolving internalized map of self and world, with a corresponding change in embodied experience Moments of innovation include a migration from one I-position to an alternative I-position, and this formed the basis of an initial theory of change	To recognize painful somatic symptoms and connect emotionally with oneself Not to be able to relate somatic troubles, emotions, and feelings	Use of externalizing problems (White and Epston, 1990) to mobilize other I positions Observing the novelty of I positions. Offering the client a language to make sense of the innovation Framing the innovation as part of a change process
DiMaggio et al. (2003) (Italy)	The self is a narrative that arises out of a relationship between characters negotiating the meaning of events with each other, with one of them emerging as the dominant one and taking control of the action Patients describe their subjective experiences by narrating stories	Intersubjectivity is expressed as partners in dialogue pursuing a common goal	The patient's negative emotions diminish in intensity, and dependent characters, suffering from guilt feelings, give way in the texts to narratives in which the self-acts independently and without feeling it is harming others if it accomplishes its own objectives	The self is able to narrate itself and admit voices that allow new meanings, writing and narrating a means to achieve it	Psychotherapists are influential in the modification of the dialogue that takes place between the characters inhabiting a patient's self. This rewriting takes place on two levels: in the patients' mind and in the dialogue between the patient and therapist
Mellado et al. (2022a) (Chile)	The self is composed of various I-positions or self-states. These self-states interact continuously as individuals engage in internal and external dialogues. A personal position is one of the possible states of the self, influenced by individual background and subject to change from one moment to another	The others are presented in a double protagonist position, internal and external, that is constituted during the history of interpersonal relationships and that situates them as characters that form part of the self in an inner scenario, influencing the subjective dynamics	Dysfunctional elements of the self can be changed as part of a repertoire of broader, organized positions. Functional parts of the self can facilitate the modification of dysfunctional parts and enable their inclusion as components of broader and more adaptive coalitions The notion of therapeutic change is a transformation of patients' subjective theories about themselves, their relationship to the environment, and their problems	Voices of the self that hinder or facilitate dialogue with the patient	Recognizing possible moments of change or enhancing therapeutic conversations from positions that are related to the change of the patient. Self-recognition and partial recognition of personal positions may be possible to the extent that patterns have already been identified and established at relevant moments in psychotherapy
Mellado et al. (2022b) (Chile)	As self-narrated in different voices, narrated stories emerge every time subjective positions are endowed with these voices, which can engage in dialogical interactions that give them dynamism	The creation of meanings is not understood as the production of particular content but rather is subject to a structure based on the activation of an intersubjective space that delimits the possibility of certain contents and meanings	A process of subjective transformation from a monological to a dialogical dimension, a more fluid exchange among the different voices of the self. A transition from a state of dissociation to a reorganization of subjectivity	Positive outcomes of the therapy are associated with the quality of the voice and position patterns that are formed in the process, which sustain these changes through the particular subjective structure that was consolidated.	Promote the construction of dialogical spaces (integrating the different positions) and the development of a reflective metaposition Talking about what has not been talked about before, reflecting on what she is talking about, and reducing monological interactions, allowing dialogical interactions to be achieved
Steen et al. (2023) (Netherlands)	Organized aspects of self-knowledge into an overarching autobiographical narrative	Respect for others and a desire for contact and union with others are characterized by care, love, intimacy, and tenderness Furthermore, visions of the good related to recognition of the self can be understood in terms of so-called S-motives, which reflect the desire for self-enhancement. In stories that people tell about themselves	To reconstruct their life narratives to become richer and more flexible, thus increasing their sense of agency and purpose Change is a recognition of the self and respect for others, and it is how being vulnerable becomes more valuable	Notions about “the good” are oriented toward meaning in life versus notions of self-blaming and disorienting purpose	Improved interpersonal functioning by identifying aspects of life and relationships as “good” through interventions by counselors
Dawson et al. (2021) (Australia)	Articulation of internal and external voices that are arranged in scenarios of greater or lesser positional hierarchy	Negotiation of silenced and blocked voices in relational scenarios of openness, closeness, and authentic listening	Renegotiation of notions of competence and collaboration in meaningful listening settings, different and differentiable from previous experiences with other health and social services	Re-signifying power relations in a web of horizontally arranged voices	Open dialogue guidelines (Seikkula et al., 2003)
Kay et al. (2021) (UK)	Internal I-positions, external I-positions, and their interactions	There is a permanent interrelation between polyphony and multivocality, attending to external and internal voices that stress mutual assemblages and resonances	The origins and changes in the relationship between internal and external I-positions	Facilitating the transit of I-positions by identifying each of these positions in each context of psychosocial application	Not made explicit
Råbu et al. (2019) (Norway)	The self is understood as a constellation of narrative accounts of one's own experiences in specific situations of personal and professional development	Intersubjectivity is understood as a collective autoethnography in which lived and enhanced experiences are made available to others through personal and collective storytelling	Change takes place through subtle learning processes that are set to establish the link between having been a client and working as a therapist, i.e., I-position, as a subjective and reflexive process of reworking meanings in the search for professional sensitivity	Facilitators and hindrances are in different areas of learning: negative experiences reinforce one's own convictions to act differently; positive experiences serve as inspiration and support; coming to therapy early in life represents an important formative experience; working through complex personal problems in therapy brings value in identifying similar conflicts	Learning transfer strategies and subjective and reflective processes of re-elaboration
Penttinen et al. (2016) (Finland)	Different positions of the self may emerge, among which may be metapositions that adopt the perspective of an author who observes the other positions and how they function as actors in certain circumstances. This dynamic allows for self-awareness and the ability to make relevant connections in one's own life experiences A community of voices in which each of them can be used when necessary	The dialogical space of encounter among interpersonal positions (therapist and patient) can be defined according to the Vygotskian concept of zone of proximal development (ZPD) (Leiman and Stiles, 2001)	Increase in patients' reflexivity and assimilation of their problematic experiences. Reflexivity at the first level allows an observational stance of one's own experiences or internal processes, and at the second level, observations about one's own way of interpreting experiences Problematic voices are accepted and integrated into the dominant community of voices	Assess patients' ability to accept the point of view being offered; assess the particular stage of the assimilation process each patient is in; Support all voices in the community. The opposite may be understood as a barrier	The therapist's responsiveness and work within the zone of proximal development (ZPD) regarding the process of assimilating patients' voices Formulation of patients' problematic experiences, empathic listening, empathetic expression, clarification, reconstruction, and gentle confrontation regarding issues that need to be constructed by the patients and their problematic experiences
Cardoso et al. (2016) (Portugal)	Development of symbolic representations of internal (including the self) and external experience. Construction of meanings from new understandings through the articulation of micronarratives and the development of macronarratives	It cannot be inferred	Coherent meaning construction is based on the interconnection and articulation of life experiences The emergence of a new narrative order and the transformation of the self	Stimulation of reflective operations that enable the construction of a coherent and continuous narrative. These can be understood as barriers, but they are the opposite	Tasks aimed at exploring patients' emotional experiences to facilitate their symbolic representation Practices that help patients connect feelings, behaviors, and life episodes promote a better understanding of the causes and consequences of their issues
Piazza-Bonin et al. (2016) (USA)	The internal world is shaped by self-narratives, which are overall cognitive-affective behavioral structures that organize daily micronarratives into macro-narratives that consolidate the understanding of the self	It cannot be inferred	The transition from narratives saturated with problems to broader, more flexible, and adaptive ones Formation and reconstruction of self-narratives	Identification, elaboration, and expansion of new events in the patients' self-narratives; reconceptualization and formation of a meta-position that makes the processes of change conscious; there is a need for affirmation and empowerment. Short-term psychotherapies can be considered barriers	Interventions for meaning generation Balance between supportive and challenging techniques so that new experiences can be better tolerated, considered, and integrated. Not to exceed the Zone of Proximal Development (ZPD) in psychotherapy
Boothe et al. (2010) (Switzerland)	Subjectivity refers to the individual's personal perspective, experiences, feelings, and interpretations that shape the narrative account. The patient's narrative is a reflection of their own subjective experience and understanding of their thoughts, emotions, and interactions	An interaction and mutual understanding between the patient and the therapist, where both parties engage in reflecting upon and exploring the patient's narrative in the context of their shared therapeutic experience	Psychological change in this context encompasses the evolution of the narrator's thoughts, emotions, and strategies as they navigate their inner conflicts and interact with external influences, ultimately leading to shifts in their understanding of themselves and their situation	Evolution of initiative: The change in initiative control from others to the ego figure indicates a potentially positive change Narrative centralization: The ego figure's transition to a central position in the narratives during the final phase suggests increased self-expression and ownership of the stories Oedipal themes and anxieties: The presence of oedipal wish and anxiety themes, as well as anxieties related to steering and control, can be challenging for the narrator Defense mechanisms: The continued use of defense mechanisms like identification with the aggressor, rationalization, and intellectualization suggests resistance to deeper emotional processing Power and autonomy axes: The continued emphasis on power and autonomy, along with a relatively unimportant closeness axis, might reflect challenges in forming close, trusting relationships	The analysis of multiple narratives and the identification of wish/anxiety/defense patterns Exploration of changes in narratives: The change in the centralization of the ego figure in the narratives Exploration of conflict themes: the identification of wish themes, anxiety themes, and conflict dynamics to identify and explore underlying conflict themes in their emotional experience Alignment of self-concept: the identification of changes in the power and autonomy axes Consideration of significant relationships: The focus is on relationships with other actors in the narratives
Danner et al. (2007) (USA)	Subjectivity refers to personal experiences, thoughts, and emotions. The Hmong women's subjective experiences of illness and healing are influenced by their cultural background. Descriptions of dreams, fears, and hopes for healing, unique perspectives on their mental health struggles, and understanding of their condition	Intersubjectivity refers to the sharing of subjective experiences and meanings, especially within a cultural context. The concept of “soul loss” or “fate” causing illness highlights a shared cultural understanding that contributes to how Hmong women perceive their mental health issues	The concept of change was referred to as overcoming psychological distress and cultural adaptation. The goal of the intervention was to facilitate a process of change that alleviates symptoms of depression, anxiety, and posttraumatic stress disorder while enhancing social support networks and coping strategies	Connection and social support: The therapy intervention created a space for connecting with others, sharing experiences, fostering understanding, and providing emotional support Learning and coping skills: Participants gained valuable coping skills through relaxation techniques, Tai Chi, and other activities. These skills empower them to manage their symptoms, reduce stress, and improve their wellbeing Practical support: Assistance with practical matters, such as help with citizenship applications and coordination with social service agencies Provider relationship: Participants established a positive and supportive relationship with the therapy facilitators, fostering trust, hope, and a sense of guidance Commitment to family and support systems: Participants found strength in their commitment to their families and children, as well as in their religious faith, providing a sense of purpose and resilience against challenging thoughts Limited transfer of skills: struggles to apply the skills to daily lives outside of the therapy sessions Persistent depression: Many participants continued to struggle with persistent depression and physical symptoms, indicating that the intervention did not completely alleviate their distress Perceived short duration: The limited number of sessions in the therapy intervention hindered participants' ability to fully delve into their concerns and make lasting changes. Some felt that more time was needed for deeper exploration	Sharing Personal Stories: This aligns with narrative therapy's focus on helping individuals construct and reframe their life stories in a way that empowers them to overcome challenges Empowerment Through Storytelling: The participants' positive experiences in sharing their stories and listening to others' stories allow them to externalize their problems and re-author their narratives Co-Construction of Meaning: The therapists collaborate with participants to co-construct meaning and explore alternative perspectives Externalization of Problems: The therapy intervention encourages participants to view their problems as external to themselves to help them separate from their difficulties Connection and Social Support: The sense of connection among participants and their shared experiences demonstrates dialogic therapy's emphasis on the importance of relationships and social support in healing and growth Open Dialogue: The participants engage in open discussions within the therapy group, allowing them to express their thoughts and emotions openly Collaborative Exploration: The multidisciplinary team of facilitators collaborates with participants to explore various aspects of their experiences and to create a space for mutual understanding and growth External Input and Community Involvement: The involvement of various community members and experts in the therapy sessions reflects dialogic therapy's broader view of therapy, where external perspectives and contributions from the community are valued
Pote et al. (2003) (UK)	Narratives, or stories, are based on behaviors and beliefs that are constructed by language and interactions among individuals who construct reality	Intersubjectivity could be considered collaborative and dynamic	Psychological change in family dynamics	The manualization process seeks to standardize therapeutic interventions. This consistency provides a structured approach that therapists can use to target specific therapeutic goals, which contributes to the facilitation of psychological change Putting principles and theoretical concepts into a manual provides a theoretical foundation for interventions. It helps therapists navigate complex family dynamics and guide their efforts toward fostering change through systemic understanding and interactional interventions Over-prescription: a manual might become overly prescriptive and restrict therapists' creativity	Narrative Exploration*:* The importance of understanding family members' stories and narratives Cultural Context: In a narrative framework, therapists would collaborate with families to understand the cultural stories that shape their identities and relationships Co-constructed Practice: The principle emphasizes the collaborative nature of therapy. This involves engaging in meaningful conversations that lead to new meanings and perspectives, echoing the dialogical idea of co-creating new understandings Circularity and Connections: The emphasis on circular patterns of behavior and connections focuses on understanding relational dynamics and interdependencies among family members

**Table 4:** Main qualitative methodologies and techniques used in the selected studies.

**Qualitative methodologies**	**Qualitative techniques**
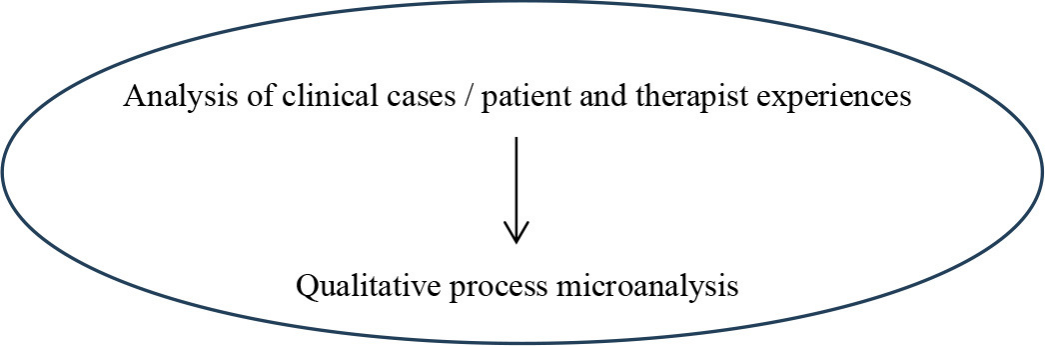 Thematic / content analysis Grounded Theory (Strauss and Corbin, 1990) Narrative analysis Discursive analysis Narrative-phenomenological analysis JAKOB narrative analysis (Boothe, 2004)	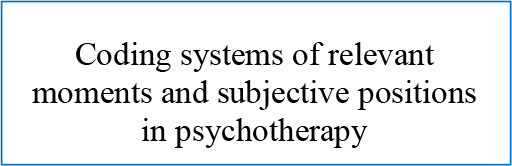 Analysis of Discursive Positioning in Psychotherapy (MAPP, Martínez and Tomicic, 2018) Innovative Moments Coding System (Gonçalves et al., 2011) Qualitative Method of Analyzing Multivoicedness (QUAM, Kay et al., 2021) 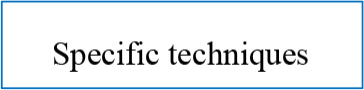 Structured, semi-structured, and open-ended interviews. Free association narrative interviews Autobiographical accounts Auto-ethnographies (therapists) Interpersonal Process Recall inquiry interviews (IPR, Kagan, 1975)

Quality assessment and risk of bias according to the MMAT criteria showed that 87.5% of the articles (*n* = 14) met >75% of the checklist criteria, indicating high quality. Twelve and a half percent of the articles (*n* = 2) met < 50% (42.86%) of the assessed criteria, indicating low quality. The assessment results are listed in Supplementary Table 1.

### 3.1 Subjectivity and intersubjectivity in psychotherapies qualitatively studied from dialogical and narrative perspectives (first review question)

From the studies examined, it can be noted that subjectivity is proposed as a dynamic and multidimensional construct involving the dialogical interaction of a multiplicity of voices and perspectives or positions (Penttinen et al., 2016; Dawson et al., 2021; Kay et al., 2021, 2023; Mellado et al., 2022a,b; Chiara et al., 2023; Hills, 2023) and that it is understood and organized through narratives that can be constantly reconstructed. These narratives reflect a unique understanding of one's personal perspective, thoughts, emotions, and interpersonal relationships. Subjectivity refers to how individuals experience and construct their sense of self and understanding of the world around them through interconnected narratives (DiMaggio et al., 2003; Boothe et al., 2010; Cardoso et al., 2016; Piazza-Bonin et al., 2016; Råbu et al., 2019; Mellado et al., 2022b; Chiara et al., 2023; Steen et al., 2023), interactions among subjective positions, and a continuous reflection on their internal and external experiences, sustained by the continuous construction of meanings (DiMaggio et al., 2003; Boothe et al., 2010: Cardoso et al., 2016; Penttinen et al., 2016; Råbu et al., 2019). It is proposed as a process in which internal voices, in constant dialogue, contribute to the formation of identity, self-awareness, a sense of agency, and the interpretation of lived experiences (Penttinen et al., 2016; Steen et al., 2023).

Self-narratives are situated as overarching structures that organize experiences into macro-narratives (more comprehensive and articulated narratives), which consolidate the understanding and meaning of the self (Cardoso et al., 2016). Subjectivity is also considered to be influenced by the cultural context, highlighting the influence of culture on the understanding of individual experiences (Danner et al., 2007).

In addition, from the information presented in some of the studies included in this review, it can be noted that intersubjectivity is proposed as a concept that underlines the relational, interactive, and collaborative basis of human experience as well as a point of connection among individuals. Intersubjectivity implies that human beings interact with each other by co-creating meanings, joint reflections, and narratives through dialogue and communication (DiMaggio et al., 2003; Danner et al., 2007; Mellado et al., 2022b; Chiara et al., 2023), generating a permanent interrelationship in which external and internal voices promote assemblies and mutual resonances (Kay et al., 2021), and positioning characters integrated into the self in an internal scenario, influencing subjective dynamics (Mellado et al., 2022a; Kay et al., 2023). Personal identities and narratives are collectively and collaboratively constructed with others through social and dialogic processes (Pote et al., 2003; Råbu et al., 2019; Chiara et al., 2023).

Intersubjectivity allows for relational unfolding in therapeutic contexts, with therapists and patients/clients collaborating to develop a trusting relationship and mutual understanding and working toward common goals in the space of encounter and recognition (Boothe et al., 2010; Penttinen et al., 2016). It is expected that in these spaces, genuine listening and openness to the negotiation of silenced and blocked voices can be generated (Dawson et al., 2021). This also involves a reciprocal process, creating an environment in which patients/clients feel free to express their experiences and emotions.

### 3.2 Psychotherapeutic change and its facilitators and barriers (second review question)

Regarding change, it has been suggested that during the therapeutic process, transformations can be observed in different dimensions of an individual's experience. These dimensions are (1) transformation and evolution of subjective internal and/or external positions and voices; an integration of voices within the self is generated, and their dysfunctional relationships, that is, rigid and inflexible repertoires, begin to be modified. Problematic or critical voices (for example, a critical I-position of a patient diagnosed with depression, *I as wronged*, expressed self-criticism based on the criticism she felt she received from “everyone” toward her) begin to be accepted and understood in a broader context, which contributes to the coherence and unity of the self (Penttinen et al., 2016; Råbu et al., 2019; Kay et al., 2021, 2023; Mellado et al., 2022a,b; Hills, 2023). (2) Transformation of narratives: transformations of personal narratives have been evidenced, along with the formation and reconstruction of self-narratives and the creation of new meanings. The re-signification of experiences and the integration of stories into personal biographies stand out as key components of change. Another relevant aspect is the construction of alternative narratives to those that were previously dominant, creating greater breadth, independence, and wellbeing (DiMaggio et al., 2003; Cardoso et al., 2016; Piazza-Bonin et al., 2016; Mellado et al., 2022a; Chiara et al., 2023; Steen et al., 2023). (3) Reconstruction of the experience of identity: individuals begin to experience changes in how they perceive themselves as well as in relation to others. A more autonomous, independent, and enriched identity develops as the self begins to re-author personal stories. Additionally, feelings of competence and self-management are generated, along with an increase in the individual's sense of agency (Dawson et al., 2021; Steen et al., 2023). (4) Reflexivity and self-observation: there is an increase in the reflexivity of patients/clients and their ability to observe and reflect on their internal experiences. Reflexivity provides an observational stance that allows individuals to more consciously question and revise interpretations of their experiences (Boothe et al., 2010; Penttinen et al., 2016). (5) Change in relationships with others: the therapeutic relationship promotes change in interpersonal relationships, serving as a model for individuals to re-signify relevant aspects of the self-other relationship and modify conflictive patterns in the different subsystems in which they interact (Pote et al., 2003; Steen et al., 2023).

Aspects that can have both a positive and negative impact on the process of change in psychotherapy should be considered.

#### 3.2.1 Change facilitators

A safe, trusting, supportive, and understanding therapeutic environment facilitates emotional and psychological change. Overall, it strengthens and supports the relational aspects of therapeutic alliances (Dawson et al., 2021). For example, Dawson et al. (2021) showed how the dialogical process of open dialogue made women who experienced domestic violence feel safe as a non-pathologizing experience in which service users defined their own issues and felt heard and validated. Participants achieved greater security and empowerment.

Focus on emotions, reflexivity, and self-reflexivity: experiencing and reflecting on emotions can enhance emotional growth and understanding of one's internal processes, which can facilitate an increase in the breadth of dialogicality and contribute to change (Cardoso et al., 2016; Kay et al., 2023). For example, Cardoso et al. (2016) described cases in which tasks were set for patients to reflexively connect feelings, behaviors, and life episodes (micro-level) to promote understanding of the causes and consequences of issues (macro-level), aiding in the subsequent construction of new meanings.

Sharing emotional experiences: shared emotional experiences can create a sense of connection and empathy, thereby creating a supportive environment of change (Danner et al., 2007; Råbu et al., 2019; Chiara et al., 2023; Hills, 2023). According to Danner et al. (2007), women from a therapeutic group managed to share their feelings and opinions about gender roles in their culture and subsequently generated alternative ideas to safely maintain contact outside the group.

Openness to new perspectives: the willingness of patients/clients to consider new ways of experiencing and thinking (new voices and positions, characters, or relationships among them that facilitate dialogue, metaperspectives, and the possibility of rewriting stories) can enable the exploration of alternatives and changes in dysfunctional thought patterns and subjective states (DiMaggio et al., 2003; Penttinen et al., 2016; Piazza-Bonin et al., 2016; Mellado et al., 2022b; Kay et al., 2023). In the therapy of a depressed patient, the external voice of her therapist joined the patient's repertoire of internal voices and helped her generate a new version of herself, incorporating an increase in her reflective capacity and willingness to dialogue with other positions. Hence, her depressive symptoms improved (Kay et al., 2023).

Strengthening patients/clients' personal resources: this refers to enquiring into and working with one's personal characteristics and dispositions (Danner et al., 2007). For example, therapists helped depressed patients connect with resources rooted in their culture (spiritual aspects that allowed them to understand health and illness) that had not been directly addressed at the beginning of treatment. This increases the legitimacy and trust placed in therapy and enables patients to identify coping strategies close to their experience (Danner et al., 2007).

Social, family, and external resource support (Pote et al., 2003; Danner et al., 2007). Pote et al. (2003) identified categories of family therapy cases, highlighting the co-construction of reality among therapists, patients, families, and their context. The family, therapeutic team, and support network promote the construction of meanings in multiple ways, avoiding the limitations of a single reality.

#### 3.2.2 Change barriers

Lack of internal dialogue: the lack of willingness to engage in internal dialogue between different parts of the self and the presence of monological patterns may hinder openness to new perspectives and the integration of experiences (Mellado et al., 2022a; Kay et al., 2023). In some patients, a “dictatorship” can be created wherein certain subjective positions are silenced, leaving them passive and voiceless. Kay et al. (2023) discussed the case of a patient in which a monological pattern dominated by a suppressive position called “I-shouldn't-exist” was identified, and this was associated with the maintenance of depressive symptoms.

Dominance of limiting or critical positions: if a limiting or highly critical position dominates one's internal dialogue, it can obstruct change by maintaining negative and self-critical patterns (Penttinen et al., 2016; Mellado et al., 2022a). A patient diagnosed with borderline personality disorder used to speak with a voice called “envious child” (identified with the Model of Discursive Positioning in Psychotherapy, MAPP) when she compared her troubled childhood with that of others. This comparison created ambivalence toward her adult life as she felt that she had not truly experienced a happy childhood (Mellado et al., 2022a).

Difficulty expressing internal experiences (intense emotions or hard-to-verbalize thoughts), rigidification of certain beliefs, and not sharing narratives in meaningful emotional contexts should be considered. Such a scenario challenges the therapeutic relationship and its understanding (Boothe et al., 2010; Chiara et al., 2023; Hills, 2023). Chiara et al. (2023) described difficulties in therapeutic changes for patients who had experienced traumatic events. Certain dominant positions (such as “I as victim”) were identified, which limited their narratives and affected their psychological growth. Progress was achieved when these positions were listened to and accepted in the therapeutic space.

External obstacles to the therapeutic process: external obstacles can hinder the therapeutic process and change. Short-term therapies with a limited number of sessions may hinder further exploration (Danner et al., 2007; Piazza-Bonin et al., 2016). The patients in one study indicated that the duration of 10 sessions of therapy was insufficient to benefit from the process (Danner et al., 2007).

### 3.3 Psychotherapeutic resources available to psychotherapists (third review question)

The types of interventions mentioned below reflect the active and collaborative role of therapists with patients/clients in the co-construction of meanings and in promoting the context of growth and subjective transformation in psychotherapy. Studies have demonstrated consistency between the theoretical and clinical assumptions of these models and the therapeutic interventions they describe.

1. Establishing empathy and acceptance: putting empathy into practice toward all voices and positions of patients/clients, allowing them to feel actively heard and understood (Penttinen et al., 2016; Chiara et al., 2023; Kay et al., 2023).
2. Co-creation or co-authoring of meaning: working with patients/clients to make sense of their experiences and explore different interpretations, perspectives, re-authoring processes, and possible meanings. Therapists can encourage the exploration of multiple perspectives and the negotiation of new storylines for narratives that arise in therapy (Pote et al., 2003; Danner et al., 2007). Using specific techniques such as externalizing problems (White and Epston, 1990), which focus on viewing difficulties as something external to the definition of the self, can mobilize different positions and perspectives (Danner et al., 2007; Hills, 2023).
3. Developing reflexivity: this involves encouraging reflection on one's own experiences and thoughts, helping patients/clients question their beliefs and perspectives, promoting the subjective and reflective processes of re-elaboration, and facilitating symbolic representation (Cardoso et al., 2016; Råbu et al., 2019). Additionally, it is crucial to explore how patients'/clients' different positions interact and influence each other and how they may change over time.
4. Promoting internal and external dialogue and the emergence of meta-positions: enable dialogue among different patient/client positions to explore internal conflicts, modify dialogues among characters that inhibit the patient's self, promote open communication, open dialogue (Seikkula et al., 2003), and build new perspectives and alternative narratives or counter-narratives (DiMaggio et al., 2003; Danner et al., 2007; Dawson et al., 2021; Mellado et al., 2022b; Chiara et al., 2023; Kay et al., 2023).
5. Amplifying change: identifying moments of change, innovations, or new perspectives in the narratives of patients/clients and exploring positions and styles of authorship that allow for their amplification and strengthening (Mellado et al., 2022a; Hills, 2023). This can facilitate the emergence of new ways of thinking and feeling as they recur in the therapeutic process.
6. Contextual interventions and analysis/use of narrative styles: working on contextualizing and reflecting on the context of the stories, including social, cultural, and personal aspects that influence the adoption of specific narratives, the analysis of the structure/coherence of autobiographical narratives, and the exploration of changes, conflict themes, and significant relationships in the narratives (Pote et al., 2003; Boothe et al., 2010; Steen et al., 2023).
7. Aligning techniques with the stage of the change process: using techniques that fit the stage of change in which patients/clients find themselves, respect their ability to address particular issues according to their subjective constitution at the moment (Piazza-Bonin et al., 2016).

## 4 Discussion

This systematic review, guided by the PRISMA guidelines, aimed to understand how subjectivity and intersubjectivity are understood in psychotherapy from dialogical and narrative approaches, as well as the process of change in psychotherapy and identify its facilitators and barriers. In addition, it provides information on psychotherapeutic resources and strategies that may be available to psychotherapists based on these approaches. For this purpose, an analysis was centered on WoS databases, which allowed us to focus on the search according to the document type, research area, and language of the eligible articles. The second phase was oriented toward the final selection of articles based on qualitative and mixed-methods empirical studies. Sixteen articles were reviewed after applying the inclusion and exclusion criteria.

This review highlights the emphasis on the transformation of meanings through dialogue and the co-creation of narratives within an intersubjective framework promoted by dialogical and narrative perspectives. The studies employed mostly qualitative methodologies that capture both narrative-dialogical exchanges and the construction of stories related to the therapeutic process, providing relevant study material for psychotherapists and those working in clinical psychology. This was also manifested in the psychotherapeutic interventions derived from previous studies, offering insights and specific tools for improving practices.

In general, the outcomes of the patients/clients reviewed in the studies highlighted awareness, self-reflection, and reflective dialogues of internal and external positions in therapy, along with a review of life experiences, as well as the co-construction of narratives in therapy. Additionally, the therapists' self-reflection processes regarding their role, functions, possibilities, and limitations were emphasized, occasionally stressing a non-expert stance and being an engaged interlocutor in a dialogue whose mission is to promote agency, subjective transformation, and empowerment of patients/clients. The dialogical approach emphasizes the exploration of patient resources, with therapists acting as active interlocutors, facilitating dialogue. The use of resources and a sense of agency enable the interplay between the elaboration of new meaning and narrative construction.

This review showed that the organization of narratives is dynamic and becomes more complex as new subjective positions are integrated through dialogical exchanges promoted by psychotherapy. In this context, the therapist's role in promoting new voices or alternative positions (Mellado et al., 2022b; Chiara et al., 2023) or offering other perspectives regarding the dominant viewpoint (or dominant voices) of patients/clients (DiMaggio et al., 2003; Kay et al., 2021, 2023) is fundamental for the mobilization of narratives. These approaches are based on the premise that patients and clients have the capacity to construct their reality and reconstruct it through reflection and the affective connections established in their social exchanges, with therapeutic relationships being a particular social instance for promoting this creative potential.

One study suggests that the Vygotskian concept of the Zone of Proximal Development (ZDP) can be understood as a dialogic space of encounter between therapist and patient positions (Penttinen et al., 2016), beyond the precautions needed when conducting psychotherapy interventions (Piazza-Bonin et al., 2016). The classical definition of ZDP indicates a space for potential learning that lies between what an individual can already do on their own and what they cannot yet do, even with external assistance (Vygotsky, 1978). In other words, it represents an individual's learning potential, which another individual can facilitate if it does not go beyond an unattainable level of demand.

Leiman and Stiles (2001) propose that the ZPD can describe the joint activity of the therapist and patient, as a transitional space between the patient's current level of development and the level they can reach with the help of the therapist to overcome their psychological difficulties. This application aligns with the stages of the patient's assimilation continuum to resolve problems and match therapeutic interventions (Zonzi et al., 2014). Encounters and mismatches can either facilitate or hinder unidirectional therapeutic progress.

Discursive and linguistic interventions guided by dialogical principles both facilitate and challenge the transition between a patient's current achievements and their potential by inquiring into each other and generating alternative meanings that are not autonomously explored or externalized. Polyphony can be an alternative for facilitating and scaffolding therapeutic changes.

Regarding some methodological tools used in the studies, Hills (2023) proposed an autoethnographic account using tools such as supervision, personal therapy, dreams, and daily life. He stated that theories of change in the therapist respond to ongoing meaning processing in the therapeutic space as dialogue evolves. He intended to demonstrate the use of self-reflection as a research instrument. Råbu et al. (2019) referred to two important processes when employing autoethnography: the therapist's self-awareness as an ongoing process understood as curiosity toward oneself and the therapy process itself, and the fact that retelling personal experiences is recognizing self-awareness. The use of personal experiences can be a threat to less experienced therapists as it inevitably triggers personal memories. In addition, having the experience of undergoing therapy was considered important for understanding clients' feelings and difficulties in relation to psychotherapy. It could be determined upon reflection at this point that to understand the client's experience, the therapist must necessarily find some common ground, be it a feeling, some knowledge, curiosity, or a familiar situation, to make sense of the process. Both Hills (2023) and Råbu et al. (2019) agreed that active self-reflection by therapists promotes therapeutic change.

In clinical practice employing narrative and dialogical approaches, therapists are considered partners in an ongoing dialogue, distinguishing their expertise as following, encouraging, and sustaining new meanings for old problems (Danner et al., 2007). This calls for respect for others, great flexibility, a sense of timing and trust, and respecting the pace and idiosyncratic ways of patients (Dawson et al., 2021). Dialogues evolve, and narratives are repeatedly told until a novel perspective is gained. Several qualities are frequently mentioned as essential to clinical practice, such as flexibility, emphatic disposition, willingness to contribute to narratives and dialogues, and promotion of new narratives (Pote et al., 2003; Penttinen et al., 2016; Piazza-Bonin et al., 2016). To this end, it should add tolerance to uncertainty, ambivalence, and ambiguity, all semiotic qualities that accompany any psychological elaboration. They are part of the manner in which the self is expressing itself, provide the stance to reframe the ongoing processing in psychotherapy, and provide an opportunity for psychotherapists to intervene. Finally, when narrating or engaging in dialogue, therapists situate themselves within a basic trust frame (Marková and Gillespie, 2007). Pieces of experiences and thoughts brought about by internal and external visiting voices were offered to both the therapist and patients. When citing Bakjtin, Markova stated that trust is vital for communication (Marková and Gillespie, 2007). Any attempt to avoid commitment and responsibility results in non-communication. Orange (2009) added that pursuing hermeneutic psychotherapeutic sensibility requires a strong awareness of one's own limitations and resources, a historical sense of the lives of others, expectations of complexity, and a strong commitment.

An important limitation of the present review lies in the deliberate search for studies that have explicitly stated the term “qualitative” to refer to the methodology used in their research, potentially excluding articles that have not declared this. In addition, most of the participants identified were psychiatric patients and individual case studies, which limits the possibilities for understanding other dimensions of these approaches (e.g., family therapy, couple therapy, group therapy, and psychosocial interventions).

Avdi and Georgaca (2007) suggest that sociocultural aspects might not be sufficiently incorporated when conducting microtherapeutic processing. This poses a limitation that should be considered when implementing microanalytical qualitative methodologies such as those used in the studies included in this review. If voices are partially shaped by culture and respond to external demands, these voices, whether internal or external, would be continually related to various external sources, as well as discursive and linguistic interventions that both facilitate and tension the generation of alternative meanings that are yet unexplored and that refer to cultural aspects that need to be considered.

Marková (2003) has emphasized that, from a Bakhtinian perspective, dialogical intersubjectivity implies providing a space for the expression of the self; that is, the individual needs the other's otherness to define themselves and simultaneously propose something new in the relationship (“I-Other(s)” irreducible dyad). Following Bakhtin (2000), if words are actions, the responsiveness and protagonism of the other allow for the unfolding of the self. Although all studies acknowledge and reinforce the notion of dialogicity in both patients and therapists, the inter-subjective framework that facilitates this process is not always evident. While not explicitly stated in all of the reviewed articles, they share a perspective on subjectivity and intersubjectivity. However, the connections between these concepts are often not clearly articulated.

Moreover, terms such as meaning construction, meaning-making processes, and narrative creation have not yet been fully explained in their conceptual and clinical scopes when approached methodologically from these perspectives. On one hand, dialogical elaboration implies active interlocutors in a constant construction, an exchange in the here and now, and possibly a basis for the formation of narratives. On the other hand, narrative creation must be defined as the result of dialogical processes in which temporally framed and meaningful narratives emerge in discourse. This entails considering not only a narrator but also multiple co-authorships, achieving the coherence of events linked to a temporally sequenced theme (White and Morgan, 2006). It could also be argued that patients/clients' narratives about their problems, as well as their drawings and written texts, trigger dialogical exchange in therapy. Thus, the construction of meanings involves evolution and movement in terms of constant elaboration with no pre-established endpoint. Narratives are constructed in a dialogical exchange mediated by cultural signs, implying the early construction of notions and meaning complexes that emerge in relationships with others. Intersubjective psychoanalytic authors point out that individuals are shaped by relatively stable patterns of relationships established early in their lives (Orange et al., 2015), which can be visualized as leading voices insofar as they can offer life perspectives and organize narratives.

Future research in the field could encompass other subjective and intersubjective dimensions in dyadic psychotherapies, including some of those already addressed in studies of family and couples therapy, among others. In this scenario, multivocality may pose a challenge for both individual and interindividual research, involving processes of meaning construction in narratives and integrating the internal and external dialogues of therapists and patients/clients. However, exploration can continue regarding how certain subjective positions are more likely to be accepted by patients/clients in accordance with the potential zones established in therapist/patient dialogical exchanges, as suggested by Leiman and Stiles (2001), Penttinen et al. (2016), Piazza-Bonin et al. (2016), and Valsiner (2007). This can be analyzed through micro-processes that account for meaning construction within changing contexts in temporally bounded segments (del Río et al., 2023) in which relatively stable bonding qualities exist. Once these constructions of meaning are identified, therapists can recognize the facilitating contexts in which the emergence of subjective positions in emotionally difficult situations can be promoted.

Regarding internal conversations, it has been proposed that there are different positions in the therapist's self that engage in dialogue with each other (Rober, 2010) as well as a continuous dialogue between the professional and personal selves of therapists (Mikes-Liu et al., 2016), which underlies some interventions that emerge in external conversations with patients. Understanding the intersection between the two dimensions can be achieved by combining descriptive methodologies, such as systems for coding subjective positions (e.g., Kay et al., 2021, 2023), with techniques that allow the recognition and tracking of internal voices, such as autoethnography (e.g., Råbu et al., 2019; Hills, 2023), or post-session semi-structured interviews with therapists and patients. In this way, greater awareness could be gained regarding how internal and external dialogues contribute and intertwine to generate transformations in the narratives of patients/clients.

## 5 Conclusion

This systematic review included a search for qualitative and mixed-methods studies on narrative and dialogical psychotherapy approaches. It encompassed topics related to subjectivity, intersubjectivity, therapeutic changes, facilitators, barriers, and intervention strategies. The analysis was based on articles published in journals indexed in JCR-WoS. The results were derived from a rigorous evaluation procedure that enabled a narrative synthesis and discussion.

The findings of this review highlight how narrative and dialogical approaches emphasize the exploration of patient resources and the promotion of reflexivity, with therapists as active interlocutors fostering therapeutic change. Moreover, tensions related to the conceptual foundations that these perspectives support were identified, suggesting the potential applications of specific psychological concepts to dialogical and narrative-oriented psychotherapy. Among other concepts, multivoicedness remains an area to be explored as a contributing aspect of psychotherapeutic practice. These insights contribute to a more comprehensive understanding of psychotherapeutic practices and their impact on patient wellbeing.

## Author contributions

AM: Conceptualization, Formal analysis, Methodology, Writing – original draft, Writing – review & editing. MR: Conceptualization, Formal analysis, Writing – original draft, Writing – review & editing. PA-A: Formal analysis, Writing – review & editing. MM: Formal analysis, Writing – review & editing.
