# Supplementary material for: Psychotherapy focusing on dialogical and narrative perspectives: a systematic review from qualitative and mixed-methods studies
Source: Front Psychol. 2024 Aug 6;15:1308131. doi: 10.3389/fpsyg.2024.1308131 (PMC11333309; doi:10.3389/fpsyg.2024.1308131)
Supplement: Supplementary file 1 [file Table_1.docx]

Table A. Quality Assessment of Selected Studies

| Authors, year (country) | **Category of study**  **designs** | **Methodological quality criteria** | **Responses** | | | |
| --- | --- | --- | --- | --- | --- | --- |
|  |  |  | Yes | No | Can’t tell | Comments |
| Kay, E., Gillespie, A., & Cooper, M. (2023). **(UK)** | Screening questions  (for all types) | S1. Are there clear research questions? | x |  |  |  |
|  |  | S2. Do the collected data allow to address the research questions? | x |  |  |  |
|  | Mixed methods | 1. Is there an adequate rationale for using a mixed methods design to address the research question? | x |  |  |  |
|  |  | 2. Are the different components of the study effectively integrated to answer the research question? | x |  |  |  |
|  |  | 3. Are the outputs of the integration of qualitative and quantitative components adequately interpreted? | x |  |  |  |
|  |  | 4. Are divergences and inconsistencies between quantitative and qualitative results adequately addressed? | x |  |  |  |
|  |  | 5. Do the different components of the study adhere to the quality criteria of each tradition of the methods involved? | x |  |  |  |
| Chiara, G., Romaioli, D., & Contarello, A. (2023). **(Italy)** | Screening questions  (for all types) | S1. Are there clear research questions? | x |  |  |  |
|  |  | S2. Do the collected data allow to address the research questions? | x |  |  |  |
|  | Qualitative | 1. Is the qualitative approach appropriate to answer the research question? | x |  |  |  |
|  |  | 2. Are the qualitative data collection methods adequate to address the research question? | x |  |  |  |
|  |  | 3. Are the findings adequately derived from the data? | x |  |  |  |
|  |  | 4. Is the interpretation of results sufficiently substantiated by data? | x |  |  |  |
|  |  | 5. Is there coherence between qualitative data sources, collection, analysis and interpretation? | x |  |  |  |
| Hills, J. (2023). **(UK)** | Screening questions  (for all types) | S1. Are there clear research questions? | x |  |  |  |
|  | Qualitative | S2. Do the collected data allow to address the research questions? | x |  |  |  |
|  |  | 1. Is the qualitative approach appropriate to answer the research question? | x |  |  |  |
|  |  | 2. Are the qualitative data collection methods adequate to address the research question? | x |  |  |  |
|  |  | 3. Are the findings adequately derived from the data? | x |  |  |  |
|  |  | 4. Is the interpretation of results sufficiently substantiated by data? | x |  |  |  |
|  |  | 5. Is there coherence between qualitative data sources, collection, analysis and interpretation? | x |  |  |  |
| DiMaggio, G., Salvatore, G., Azzara, C., & Catania, D. (2003). **(Italy)** | Screening questions  (for all types) | S1. Are there clear research questions? | x |  |  |  |
|  |  | S2. Do the collected data allow to address the research questions? | x |  |  |  |
|  | Mixed methods | 1. Is there an adequate rationale for using a mixed methods design to address the research question? | x |  |  |  |
|  |  | 2. Are the different components of the study effectively integrated to answer the research question? | x |  |  |  |
|  |  | 3. Are the outputs of the integration of qualitative and quantitative components adequately interpreted? | x |  |  |  |
|  |  | 4. Are divergences and inconsistencies between quantitative and qualitative results adequately addressed? | x |  |  |  |
|  |  | 5. Do the different components of the study adhere to the quality criteria of each tradition of the methods involved? |  |  | x |  |
| Mellado, A., Martínez, C., Tomicic, A. & Krause, M. (2022 a). **(Chile)** | Screening questions  (for all types) | S1. Are there clear research questions? | x |  |  |  |
|  |  | S2. Do the collected data allow to address the research questions? | x |  |  |  |
|  | Mixed methods | 1. Is there an adequate rationale for using a mixed methods design to address the research question? | x |  |  |  |
|  |  | 2. Are the different components of the study effectively integrated to answer the research question? | x |  |  |  |
|  |  | 3. Are the outputs of the integration of qualitative and quantitative components adequately interpreted? | x |  |  |  |
|  |  | 4. Are divergences and inconsistencies between quantitative and qualitative results adequately addressed? | x |  |  |  |
|  |  | 5. Do the different components of the study adhere to the quality criteria of each tradition of the methods involved? |  |  | x |  |
| Mellado, A., Martínez, C., Tomicic, A. & Krause, M. (2022 b). **(Chile)** | Screening questions  (for all types) | S1. Are there clear research questions? | x |  |  |  |
|  |  | S2. Do the collected data allow to address the research questions? | x |  |  |  |
|  | Mixed methods | 1. Is there an adequate rationale for using a mixed methods design to address the research question? | x |  |  |  |
|  |  | 2. Are the different components of the study effectively integrated to answer the research question? | x |  |  |  |
|  |  | 3. Are the outputs of the integration of qualitative and quantitative components adequately interpreted? | x |  |  |  |
|  |  | 4. Are divergences and inconsistencies between quantitative and qualitative results adequately addressed? | x |  |  |  |
|  |  | 5. Do the different components of the study adhere to the quality criteria of each tradition of the methods involved? | x |  |  |  |
| Steen, A., Graste, S., Schuhmann, C., de Kubber, S., & Braam, A. W. (2023). **(Netherlands)** | Screening questions  (for all types) | S1. Are there clear research questions? | x |  |  |  |
|  |  | S2. Do the collected data allow to address the research questions? | x |  |  |  |
|  | Mixed methods | 1. Is there an adequate rationale for using a mixed methods design to address the research question? | x |  |  |  |
|  |  | 2. Are the different components of the study effectively integrated to answer the research question? | x |  |  |  |
|  |  | 3. Are the outputs of the integration of qualitative and quantitative components adequately interpreted? | x |  |  |  |
|  |  | 4. Are divergences and inconsistencies between quantitative and qualitative results adequately addressed? | x |  |  |  |
|  |  | 5. Do the different components of the study adhere to the quality criteria of each tradition of the methods involved? |  |  | x |  |
| Dawson, L., Einboden, R., McCloughen, A., & Buus, N. (2020) **(Australia)** | Screening questions  (for all types) | S1. Are there clear research questions? | x |  |  |  |
|  |  | S2. Do the collected data allow to address the research questions? | x |  |  |  |
|  | Qualitative | 1. Is the qualitative approach appropriate to answer the research question? | x |  |  |  |
|  |  | 2. Are the qualitative data collection methods adequate to address the research question? | x |  |  |  |
|  |  | 3. Are the findings adequately derived from the data? | x |  |  |  |
|  |  | 4. Is the interpretation of results sufficiently substantiated by data? |  | x |  | The authors consider that there was no saturation of results |
|  |  | 5. Is there coherence between qualitative data sources, collection, analysis and interpretation? | x |  |  |  |
| Kay, E., Gillespie, A., & Cooper, M. (2021). **(UK)** | Screening questions  (for all types) | S1. Are there clear research questions? | x |  |  |  |
|  |  | S2. Do the collected data allow to address the research questions? | x |  |  |  |
|  | Mixed methods | 1. Is there an adequate rationale for using a mixed methods design to address the research question? | x |  |  |  |
|  |  | 2. Are the different components of the study effectively integrated to answer the research question? | x |  |  |  |
|  |  | 3. Are the outputs of the integration of qualitative and quantitative components adequately interpreted? | x |  |  |  |
|  |  | 4. Are divergences and inconsistencies between quantitative and qualitative results adequately addressed? | x |  |  |  |
|  |  | 5. Do the different components of the study adhere to the quality criteria of each tradition of the methods involved? | x |  |  |  |
| Råbu, M., McLeod, J., Haavind, H., Bernhardt, I. S., Nissen-Lie, H., & Moltu, C. (2019). **(Norway)** | Screening questions  (for all types) | S1. Are there clear research questions? | x |  |  |  |
|  |  | S2. Do the collected data allow to address the research questions? | x |  |  |  |
|  | Qualitative | 1. Is the qualitative approach appropriate to answer the research question? | x |  |  |  |
|  |  | 2. Are the qualitative data collection methods adequate to address the research question? | x |  |  |  |
|  |  | 3. Are the findings adequately derived from the data? | x |  |  |  |
|  |  | 4. Is the interpretation of results sufficiently substantiated by data? | x |  |  |  |
|  |  | 5. Is there coherence between qualitative data sources, collection, analysis and interpretation? | x |  |  |  |
| Penttinen, H., Wahlström, J., & Hartikainen, K. (2016).  **(Finlandia)** | Screening questions  (for all types) | S1. Are there clear research questions? | x |  |  | A general research question is not provided. |
|  |  | S2. Do the collected data allow to address the research questions? | x |  |  |  |
|  | Qualitative | 1. Is the qualitative approach appropriate to answer the research question? | x |  |  |  |
|  |  | 2. Are the qualitative data collection methods adequate to address the research question? | x |  |  |  |
|  |  | 3. Are the findings adequately derived from the data? | x |  |  |  |
|  |  | 4. Is the interpretation of results sufficiently substantiated by data? | x |  |  |  |
|  |  | 5. Is there coherence between qualitative data sources, collection, analysis and interpretation? | x |  |  |  |
| Cardoso, P., Duarte, M. E., Gaspar, R., Bernardo, F., Janeiro, I. N., & Santos, G. (2016).  **(Portugal)** | Screening questions  (for all types) | S1. Are there clear research questions? | x |  |  |  |
|  |  | S2. Do the collected data allow to address the research questions? | x |  |  |  |
|  | Qualitative | 1. Is the qualitative approach appropriate to answer the research question? | x |  |  |  |
|  |  | 2. Are the qualitative data collection methods adequate to address the research question? | x |  |  |  |
|  |  | 3. Are the findings adequately derived from the data? | x |  |  |  |
|  |  | 4. Is the interpretation of results sufficiently substantiated by data? | x |  |  |  |
|  |  | 5. Is there coherence between qualitative data sources, collection, analysis and interpretation? | x |  |  |  |
| Piazza-Bonin, E., Neimeyer, R. A., Alves, D., Smigelsky, M., & Crunk, E. (2016).  **(USA)** | Screening questions  (for all types) | S1. Are there clear research questions? | x |  |  |  |
|  |  | S2. Do the collected data allow to address the research questions? | x |  |  |  |
|  | Mixed methods | 1. Is there an adequate rationale for using a mixed methods design to address the research question? | x |  |  |  |
|  |  | 2. Are the different components of the study effectively integrated to answer the research question? | x |  |  |  |
|  |  | 3. Are the outputs of the integration of qualitative and quantitative components adequately interpreted? | x |  |  |  |
|  |  | 4. Are divergences and inconsistencies between quantitative and qualitative results adequately addressed? | x |  |  |  |
|  |  | 5. Do the different components of the study adhere to the quality criteria of each tradition of the methods involved? | x |  |  |  |
| Boothe, B.,  Grimm, G.,  Hermann, M.L., &  Luder, M. (2010) **(Switzerland)** | Screening questions  (for all types) | S1. Are there clear research questions? |  |  | x |  |
|  |  | S2. Do the collected data allow to address the research questions? | x |  |  |  |
|  | Qualitative | 1. Is the qualitative approach appropriate to answer the research question? | x |  |  |  |
|  |  | 2. Are the qualitative data collection methods adequate to address the research question? | x |  |  |  |
|  |  | 3. Are the findings adequately derived from the data? |  |  | x |  |
|  |  | 4. Is the interpretation of results sufficiently substantiated by data? |  |  | x |  |
|  |  | 5. Is there coherence between qualitative data sources, collection, analysis and interpretation? |  | x |  |  |
| Danner, C.C., Robinson, B.B.E., Striepe, M.I., & Rhodes, P.F.Y. (2007) **(USA)** | Screening questions  (for all types) | S1. Are there clear research questions? | x |  |  |  |
|  |  | S2. Do the collected data allow to address the research questions? | x |  |  |  |
|  | Mixed methods | 1. Is there an adequate rationale for using a mixed methods design to address the research question? | x |  |  |  |
|  |  | 2. Are the different components of the study effectively integrated to answer the research question? |  |  | x |  |
|  |  | 3. Are the outputs of the integration of qualitative and quantitative components adequately interpreted? | x |  |  |  |
|  |  | 4. Are divergences and inconsistencies between quantitative and qualitative results adequately addressed? | x |  |  |  |
|  |  | 5. Do the different components of the study adhere to the quality criteria of each tradition of the methods involved? | x |  |  |  |
| Pote, H.,  Stratton, P.,  Cottrell, D.,  Shapiro, D., &  Boston, P.  (2003) **(UK)** | Screening questions  (for all types) | S1. Are there clear research questions? | x |  |  |  |
|  |  | S2. Do the collected data allow to address the research questions? | x |  |  |  |
|  | Qualitative | 1. Is the qualitative approach appropriate to answer the research question? |  | x |  | The design is not described in depth. |
|  |  | 2. Are the qualitative data collection methods adequate to address the research question? | x |  |  |  |
|  |  | 3. Are the findings adequately derived from the data? |  |  | x | The results are general, and illustrative quotations are not provided. |
|  |  | 4. Is the interpretation of results sufficiently substantiated by data? |  |  | x |  |
|  |  | 5. Is there coherence between qualitative data sources, collection, analysis and interpretation? |  | x |  |  |

The quality assessment criteria for the studies are based on the Mixed Methods Appraisal Tool (MMAT). Reference: Hong QN, Pluye P, Fàbregues S, Bartlett G, Boardman F, Cargo M, Dagenais P, Gagnon M-P, Griffiths F, Nicolau B, O’Cathain A, Rousseau M-C, Vedel I. Mixed Methods Appraisal Tool (MMAT), version 2018. Registration of Copyright (#1148552), Canadian Intellectual Property Office, Industry Canada.
